# Electrophysiological and Behavioral Responses of *Apis mellifera* and *Bombus* *terrestris* to Melon Flower Volatiles

**DOI:** 10.3390/insects13110973

**Published:** 2022-10-22

**Authors:** Jiangchao Zhang, Jinjia Liu, Fei Gao, Min Chen, Yusuo Jiang, Huiting Zhao, Weihua Ma

**Affiliations:** 1 College of Animal Science, Shanxi Agricultural University, Taigu 030801, China; 2 College of Life Sciences, Shanxi Agricultural University, Taigu 030801, China; 3 College of Horticulture, Shanxi Agricultural University, Taiyuan 030031, China

**Keywords:** *Apis mellifera*, *Bombus terrestris*, flower volatiles, electroantennography (EAG), olfactory behavior, melon

## Abstract

**Simple Summary:**

Honeybees (*Apis mellifera*) and bumblebees (*Bombus terrestris*) are often used to pollinate melon flowers in facilities. The researchers identified the volatiles in male and female flowers of a common melon species (*Cucumis melo*) grown in facilities and measured the EAG and behavioral responses of honeybees and bumblebees when exposed to isolated volatiles from the melon flowers. These results provide basic data for the rational utilization of bees.

**Abstract:**

As important pollinators, honeybees and bumblebees present a pollination behavior that is influenced by flower volatiles through the olfactory system. In this study, volatile compounds from melon flowers were isolated and identified by headspace solid-phase microextraction (HS-SPME) and gas chromatography–mass spectrometry (GC-MS), and their effects on *Apis mellifera* and *Bombus terrestris* were investigated by electroantennogram (EAG) and behavior tests (Y-tube olfactometer). The results showed that 77 volatile compounds were detected in melon flowers, among which the relative content of aldehydes was the highest (61.34%; 82.09%). *A. mellifera* showed a strong EAG response to e-2-hexenal, e-2-octenal, and 1-nonanal. *B. terrestris* showed a strong EAG response to e-2-hexenal, e-2-octenal, 2,5-dimethyl-benzaldehyde, benzaldehyde and benzenepropanal. In behavior tests, the volatiles with the highest attractive rate to *A. mellifera* were e-2-hexenal (200 μg/μL, 33.33%) and e-2-octenal (300 μg/μL, 33.33%), and those to *B. terrestris* were e-2-hexenal (10 μg/μL, 53.33%) and 2,5-dimethyl-benzaldehyde (100 μg/μL, 43.33%). E-2-hexenal and e-2-octenal were more attractive to *A. mellifera* than *B. terrestris*, respectively (10 μg/μL, 10 μg/μL, 200 μg/μL). In conclusion, the volatiles of melon flowers in facilities have certain effects on the electrophysiology and behavior of bees, which is expected to provide theoretical and technical support for the pollination of *A. mellifera* and *B. terrestris* in facilities.

## 1. Introduction

Honeybees and bumblebees are regarded as the ideal pollinators in the world, playing an important role in improving ecological economic benefits and protecting the balance of the ecosystem [1,2,3]. They pollinate fruit and vegetable crops and play an important role in increasing yields and incomes [4,5,6].

Plant flowers regulate the interaction between pollinators and plants by releasing volatiles. Pollinators can identify volatiles of flowers according to their nutrition and reproductive needs, judge distance and location, and conduct pollen collection [7,8]. The volatile compounds of flowers have important effects on pollinator searching behavior [9,10]. Pollinators and flowering plants interact [11]. Pollinator collection relies not only on vision but also on olfactory signals. It has been found that pollinator visitors rely more on olfactory signals when visiting flowers [12,13]. Therefore, flower volatiles play a key role in the interaction between pollinators and plants.

Melon, *Cucumis melon* (*Cucurbitaceae*), is monoecious and cross-pollinated. Facility agriculture can shorten the production cycle and meet the demand for out-of-season fruits. Honeybees and bumblebees are important pollinators of facility crops. 

In this study, solid-phase microextraction (SPME) and gas chromatography–mass spectrometry (GC-MS) were used to determine the volatiles in melon flowers [14], and the volatile components with a strong tendency were screened out by electroantennogram (EAG) techniques and behavior tests, and the response of *Apis mellifera* and *Bombus terrestris* to volatiles was observed. The aim was to select suitable pollinators for melon pollination and use volatile regulation to improve the pollination effect. Moreover, studying the effects of flower volatiles on *A. mellifera* and *B. terrestris* provides more scientific guidance for crop pollination.

## 2. Materials and Methods

### 2.1. Insects

Honeybees (*A. mellifera* *ligustica* Spin.) used in the experiment came from the apiary of the College of Animal Science and Technology of Shanxi Agricultural University. Bumblebees (*B. terrestris*) were purchased from Hebei Hengshui WoFeng Biotechnology Company. Bees were collected for the EAG recording and behavioral tests at 9–10 am at the entrance of the hive by using two tweezers to capture those with obvious pollen on their legs. They were placed in a wooden box and supplied with a 50% sugar solution. 

### 2.2. Plants

The tested melon, Xing-tian melon, a local variety, was grown in greenhouses at Dongshandi Village base, Taigu County, Jinzhong City, Shanxi Province. Male and female melon flowers were collected and sent to Qingdao Kechuang Quality Testing Co., Ltd. (Qingdao, China) for volatile content determination.

### 2.3. Identification of Volatile Compounds

The collected flower was weighed 2 g and placed in a 20 mL headspace bottle, sealed with 3 mL saturated NaCl solution (Manufacturer: Sinopharm Chemical Reagent Co., Ltd., Shanghai, China), balanced at 80 °C for 30 min, and extracted with an extraction needle (Model: 100 μL PDMS fiber tip; Manufacturer: Supelco, Bellefonte, PA, USA) for 30 min. After the extraction, the extraction needle was desorbed at the injection port for 5 min. 

Gas chromatography–mass spectrometry (GC-MS) (Model: 6890N-5975B; Manufacturer: Agilent Technologies Spain, S.L., Madrid, Spain) was used for qualitative and semiquantitative analysis. The GC was equipped with a HP-5MS elastic quartz capillary column (30 m × 0.25 mm × 0.25 μm). Helium (Purity ≥ 99.999%) was used as the carrier gas at a constant flow rate of 1.0 mL/min. The inlet temperature was 240 °C, without split injection. The oven temperature was kept at 45 °C for 5 min, then it was raised to 130 °C at a rate of 6 °C/min, finally increased to 240 °C at a rate of 10 °C/min, and maintained isothermally for 8 min. For MS conditions, an electron impact (EI) ionization system was used at 70 eV in full-scan acquisition mode with a mass range of 40–450 U. The temperature of the interface was 280 °C; the temperature of the ion source was 230 °C [15]. 

Volatile compound analysis and identification were conducted using a GCMS-QP2010 Plus with Nist 107 and Wiley 229 mass spectral libraries. After removing impurities, combined with the manual retrieval of qualitative analysis components, the total ion graph peak area normalization method was used for quantitative analysis, using the equation as follows:

Area (%) = area of single compound peak × 100/area of total compound peak

### 2.4. EAG Recording

The standard compounds for EAG recording were determined according to the identification results of volatile compounds in female and male flowers of melon. The standard compounds for EAG recording are shown in Table 1. 

The antennae of *A. mellifera* and *B. terrestris* were stimulated by volatiles, and antennae potentiometers (Ockenfels Syntech GMBH, Buchenbach, Germany) were used to measure potential changes to illustrate the sensitivity of antennae to volatiles. 

A pair of bee antennas were cut with a surgical blade and connected to an electrode with conductive adhesive (SpectraR360). The electrode was connected to a DC/AC amplifier (Syntech IDAC-4), and the output end of the amplifier signal was connected to a computer. The filter paper (3 cm × 1.5 cm) was folded into a “V” shape, 10 μL of the sample to be measured was dropped onto the filter paper and put into the sample tube, and the end of the sample tube was connected with the odor stimulation control device. The antennae potentiometer stimulus gas controller (Syntech CS-55) sent out two test air streams blowing together to the separated antennae: one was a continuous air stream directly blown out after filtration and humidification, and the other was a stream carrying the pungent odor of the sample. The airflow port and antennae were vertical, about 9 mm apart. The stimulated flow was 40 mL/min and the continuous flow was 500 mL/min. 

With liquid paraffin as the solvent, the standard compound was prepared into 6 concentration gradients of 10, 100, 200, 300, 400, and 500 μg/μL to be tested, and the test was carried out with paraffin as control. Each antenna was alternately tested by liquid paraffin and sample, with the sample concentration from low to high. The antennae were replaced after one repeat of each sample, and the test was repeated 3 times in total. To prevent the antennae from being stimulated by the sample, bees should be kept away from the sample before each cutting of the antennae. The response of bees to volatiles was expressed by EAG relative response value (means ± standard error). The EAG relative response values (rEAG) are calculated as follows:

rEAG (%) = (EAG(X) − EAG(std))/EAG(std)

where EAG(X) is the amplitude (mV) of the EAG response to a compound and EAG(std) is the amplitude (mV) of the EAG response to the reference liquid paraffin of each recording session.

### 2.5. Behavior Tests

Based on EAG determination, the samples that could induce a strong reaction of the antennae of *A. mellifera* and *B. terrestris* were selected as the test compounds for olfactory behavior response (Table 1). The concentration gradient of the compounds to be measured is the same as that at the EAG recording.

Behavior experiments were carried out using a Y-tube olfactometer (Nanjing Possum Instrument Co., Ltd., Nanjing, Jiangsu, China) in a dark room. The tests were conducted between 08:00 and 12:00 am. The sequence of gas passing through the device was as follows: air pump (Beijing Kean Labor Bao New Technology Company, Beijing, China) → activated carbon drying tube → distilled water humidifying bottle → gas flow meter → volatile matter flavor source bottle → Y-tube olfactometer. The devices were connected by silica gel tubes. Then 10 μL samples and liquid paraffin (control) were added to two pieces of filter paper prepared in advance (3 × 1.5 cm^2^), and the two pieces of filter paper were placed in the flavor source bottle as the odor source, and the gas flow rate was 200 mL/min. The air pump was turned on for about 10 s to fill the Y-tube olfactometer with gas. The bees were then placed in the middle of the Y-tube olfactometer, and when the bees were familiar with the environment around the Y-tube olfactometer, the air pump was turned on again to observe the bees’ selection of smell and record it. 

The evaluation conditions of bees’ odor selection were as follows: the observation time of each collecting bee was at least 10 min, and if the collecting bee entered the odor bottle or stayed in the front 1/3 area of the odor bottle for more than 4 min, it was regarded as the selection of samples in the odor bottle. Samples and paraffin in the vials should be replaced every time they are used (5 honeybees per test). The test was repeated for 3 groups with 10 honeybees in each group. After testing two honeybees, the olfactory machine was reversed or replaced to eliminate interference. After one sample was tested, the odor bottle and olfactory apparatus were cleaned with 75% ethanol and distilled water to eliminate residual odors for future use. 

The results of behavioral tests were expressed by the repellent rate and attractive rate. The following equation is used to calculate the attractive rate and the repellent rate:

Attractive Rate (%) = total number of bees in treatment arm/total number of bees tested × 100%

Repellent Rate (%) = total number of bees in control arm/total number of bees tested × 100%

### 2.6. Statistical Analysis

EAG relative response values (rEAG), attractive rate, and repellent rate were calculated by the formula as described in the Method section. The results of EAG tests were analyzed by one-way ANOVA, Fisher’s protected least-significant-difference (LSD) multiple-comparison procedure was used to analyze the EAG response of *A. mellifera* and *B. terrestris* to volatiles at different concentrations. Independent sample t-test was used to analyze the difference in EAG response among different volatiles at the same concentration and different bee species. The results of behavioral tests were analyzed by the chi-square test. SPSS21 was used for data analysis, and Excel and GraphPad5.0 were used for drawing.

## 3. Results

### 3.1. Identification of Volatile Compounds

The results of volatile compound identification showed that 77 volatiles were detected in the female and male flowers of melon, including aldehyde, alkane, ether, alkyne, alkene, ester, alcohol, aromatics, ketone, and others (Table 2). There were similarities and differences in the types and contents of volatiles in flowers. 

**Figure 1:**
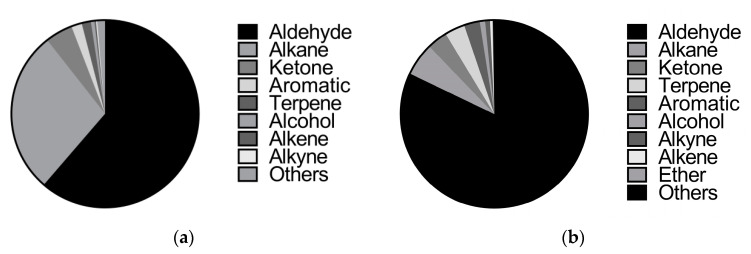
Proportion of volatile species in female and male flowers of melon. (**a**) Analysis of the proportion of volatile species in female flowers of melon; (**b**) analysis of the proportion of volatile species in male flowers of melon.

The volatiles of female melon flowers contained 39 compounds in 9 groups. There were 9 aldehydes (61.34%), 5 alkanes (28.08%), 7 ketones (4.75%), 5 aromatics (1.82%), 4 esters (1.59%), 4 alcohols (0.61%), 1 alkene (0.27%), 2 alkynes (0.21%), and 2 other compounds (1.35%). The volatiles of male melon flowers contained 52 compounds in 10 groups. There were 13 aldehydes (82.09%), 3 alkanes (5.69%), 9 ketones (3.51%), 9 esters (3.48%), 7 aromatics (2.72%), 6 alcohols (0.92%), 1 alkyne (0.85%), 1 alkene (0.38%), 1 ether (0.12%), and 2 other compounds (0.24%) (Table 2, Figure 1).

Fifteen compounds were detected in both female and male flowers of melon. The relative contents of these volatiles accounted for 64.87% and 83.02% of the total volatiles in female and male flowers, respectively. Benzaldehyde had the highest relative content in both female and male flowers of melon (48.83% and 74.39%, respectively) (Table 3, Figure 1). 

### 3.2. EAG Response of Bees to Volatiles

#### 3.2.1. EAG Response of *A. mellifera*

*A. mellifera* had different rEAG of the same volatiles in different concentrations. The rEAG of *A. mellifera* to 3-phenyl-2-propenal and e-2-decenal at high concentrations were significantly higher than that at low concentrations (*p* < 0.05). The rEAG of dimethyl phthalate and heptadecane at low concentrations were significantly higher than that at high concentrations (*p* < 0.05). The rEAG of benzaldehyde and e-2-hexenal showed an increasing trend and then decreased with the increase in the concentration. For 1-nonanal and phytol, their rEAG increased with the increase in the concentration and reached the maximum (Table A1). 

For *A. mellifera*, there were some volatiles with high rEAG, including 1-nonanal, e-2-hexenal, e-2-octenal, and benzaldehyde. The rEAG of *A. mellifera* to 1-nonanal was the highest among all the concentrations. At the concentration of 10 μg/μL, the rEAG of 1-nonanal was significantly higher than that of phytol (*p* < 0.05). At the concentration of 100 μg/μL to 300 μg/μL, the rEAG of 1-nonanal was significantly higher than that of e-2-hexenal and benzaldehyde (*p* < 0.05). (Table A1).

#### 3.2.2. EAG Response of *B. terrestris*

*B. terrestris* had different rEAGs of the same volatiles in different concentrations. The rEAGs of 2,5-dimethyl-benzaldehyde, 1,2-dimethoxy-4-(1-propenyl)-benzene, and dodecanal at high concentrations were significantly higher than those at low concentrations (*p* < 0.05). The rEAG of 1-nonanal increased with the increase in the concentration and reached the maximum when the concentration was 400 μg/μL (Table A2).

For *B. terrestris*, there were some volatiles with higher rEAG, including benzaldehyde, e-2-octenal, e-2-hexenal, and benzenepropanal. At the concentration of 10 μg/μL, benzaldehyde was significantly higher than that of dimethyl phthalate (*p* < 0.05). At the concentration of 100 and 500 μg/μL, the rEAG of e-2-octenal was significantly higher than those of benzenepropanal and e-2-hexenal (*p* < 0.05). At the concentration of 200 μg/μL to 400 μg/μL, the rEAG of e-2-octenal was significantly higher than that of benzenepropanal (*p* < 0.05) (Table A2).

#### 3.2.3. Comparison of EAG responses between *A. mellifera* and *B. terrestris*

**Figure 2:**
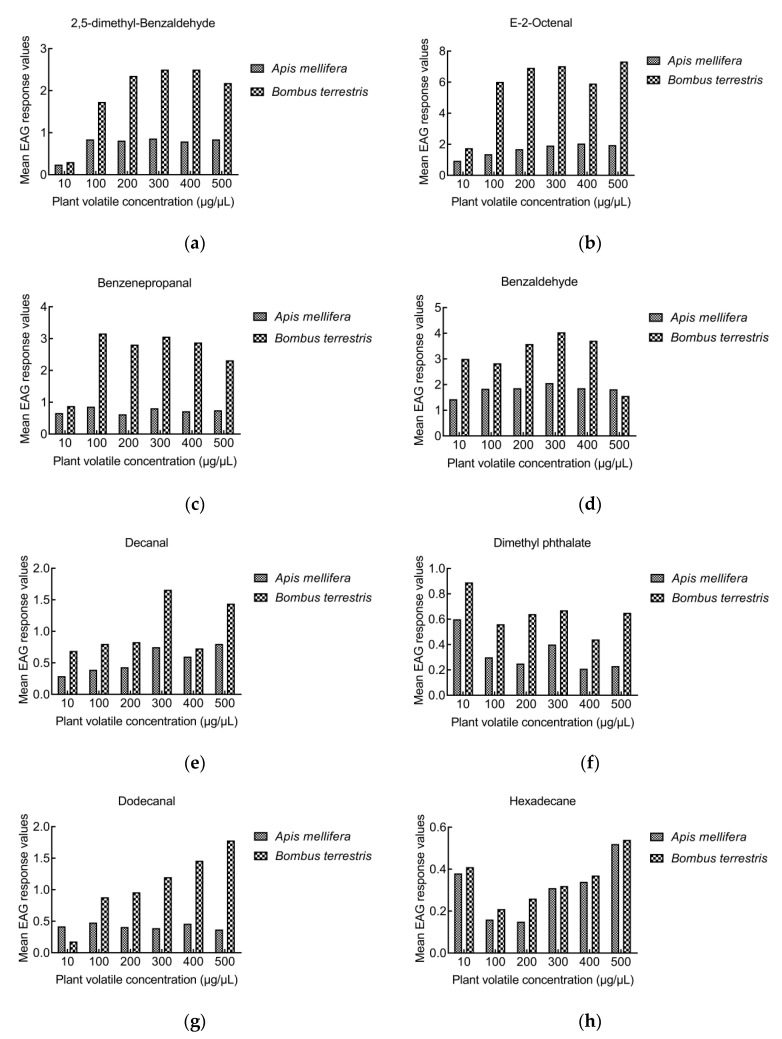
Comparison of EAG responses between *A. mellifera* and *B. terrestris*. The rEAGs of *B. terrestris* higher than those of *A. mellifera*: (**a**) 2,5-dimethyl-benzaldehyde, (**b**) e-2-octenal, (**c**) benzenepropanal, (**d**) benzaldehyde, (**e**) decanal, (**f**) dimethyl phthalate, (**g**) dodecanal, (**h**) hexadecane.

**Figure 3:**
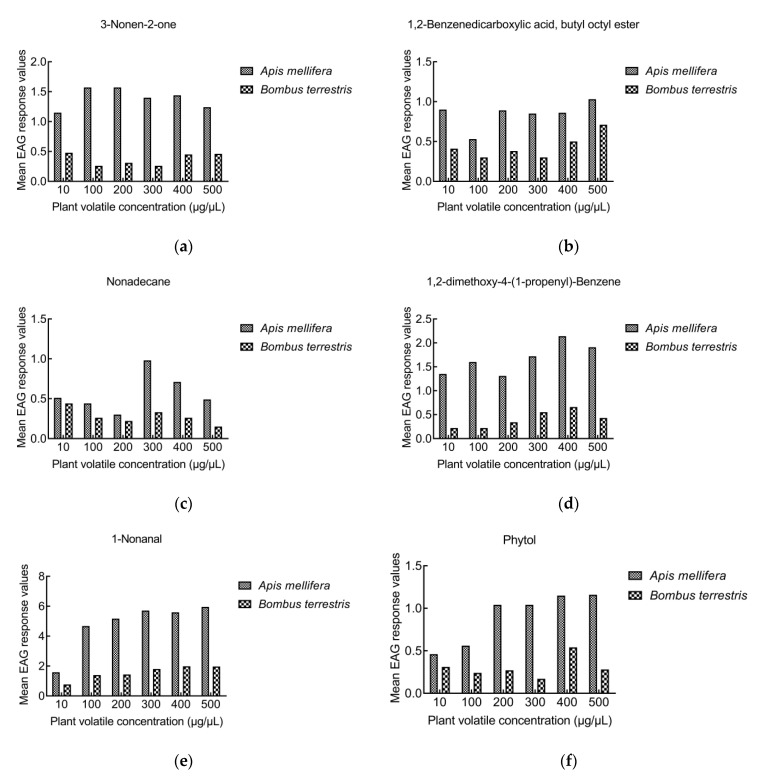
Comparison of EAG responses between *A. mellifera* and *B. terrestris*. The rEAGs of *A. mellifera* are higher than those of *B. terrestris*: (**a**) 3-nonen-2-one, (**b**) 1,2-benzene dicarboxylic acid, butyl octyl ester, (**c**) nonadecane, (**d**) 1,2-dimethoxy-4-(1-propenyl)-benzene, (**e**) 1-nonanal, (**f**) phytol.

When bees were stimulated with the same concentration of volatiles, the rEAG of *B. terrestris* to e-2-octenal, benzenepropanal, hexadecane, 2,5-dimethyl-benzaldehyde, benzaldehyde, dimethyl phthalate, dodecanal, and decanal were higher than those of *A. mellifera*. However, the rEAGs of *A. mellifera* to 1-nonanal, 3-nonen-2-one, nonadecane, phytol, 1,2-dimethoxy-4-(1-propenyl)-benzene, and 1,2-benzene dicarboxylic acid, butyl octyl ester were higher than those of *B. terrestris* (Figure 2 and Figure 3). The concentration of volatiles that caused the strong EAG reaction was mainly concentrated at 500 μg/μL in *A. mellifera*, but at 400 μg/μL in *B. terrestris*.

**Figure 4:**
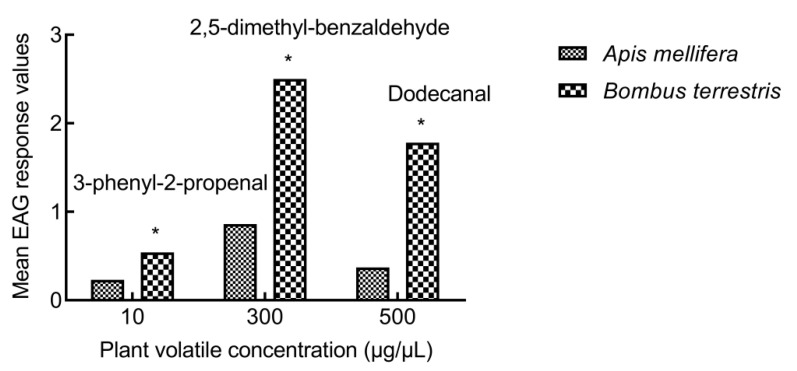
Comparison of EAG responses between *A. mellifera* and *B. terrestris* to 3-phenlyl-2-propenal, 2,5-dimethyl-benzaldehyde, and dodecanal. “*” means significant difference (*p* < 0.05).

The rEAG of *A. mellifera* to 1-nonanal was the highest among all the concentrations. At the concentration of 10 μg/μL, the rEAG of *B. terrestris* to 3-phenyl-2-propenal was significantly higher than that of *A. mellifera*. At the concentration of 300 μg/μL, the rEAG of *B. terrestris* to 2,5-dimethyl-benzaldehyde was significantly higher than that of *A. mellifera*. At the concentration of 500 μg/μL, the rEAG of *B. terrestris* to dodecanal was significantly higher than that of *A. mellifera*. For other compounds, there were no significant differences between the two bee species (Figure 4).

### 3.3. Behavior Tests

#### 3.3.1. Behavior Tests of *A. mellifera*

According to the EAG response test results of *A. mellifera*, three compounds with higher relative reaction values of EAG were selected for the behavior test: e-2-hexenal, e-2-octenal, and 1-nonanal. 

**Figure 5:**
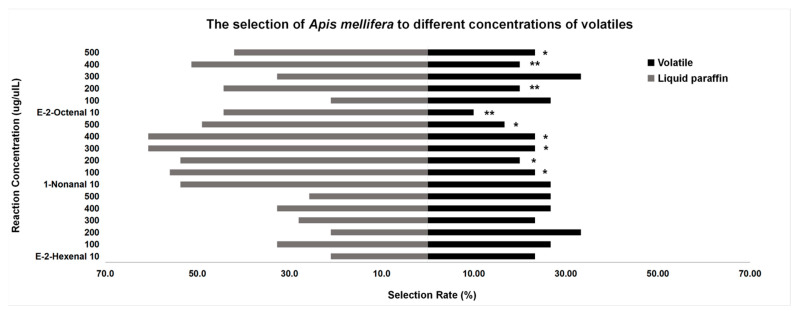
Behavior tests of A. mellifera. “*” means significant difference (*p* < 0.05), “**” means extremely significant difference (*p* < 0.01).

At the concentration of 200 μg/μL, the attractive rate of e-2-hexenal to *A. mellifera* (33.33%) was higher than the repellent rate (30.00%). In the concentration of e-2-octenal at 10 μg/μL, 200 μg/μL, 400 μg/μL, and 500 μg/μL, *A. mellifera* preferred liquid paraffin (*p* < 0.05). Compared with 1-nonanal, *A. mellifera* preferred liquid paraffin (*p* < 0.05), but there was no significant difference at 10 μg/μL (*p* > 0.05). At the same concentration of different volatiles, from 10 to 500 μg/μL, the volatiles with the highest attractive rate to honeybees were: 1-nonanal (26.67%), e-2-hexenal and e-2-octenal (26.67%), e-2-hexenal (33.33%), e-2-octenal (33.33%), e-2-hexenal (26.67%), and e-2-hexenal (26.67%) (Figure 5).

#### 3.3.2. Behavior Tests of *B. terrestris*

According to the EAG response test results of *B. terrestris*, five compounds with higher relative reaction values of EAG were selected for the behavior test: e-2-hexenal, e-2-octenal, benzaldehyde, 2,5-dimethyl-benzaldehyde, and benzenepropanal.

**Figure 6:**
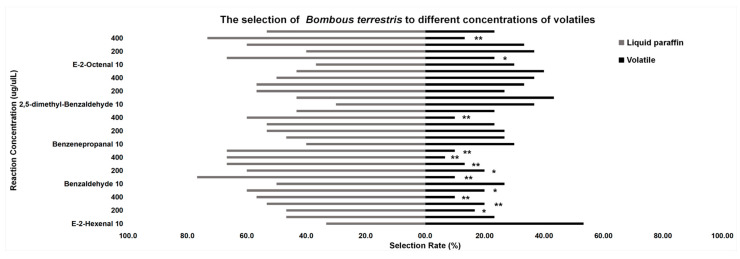
Behavior tests of *B. terrestris*. “*” means significant difference (*p* < 0.05), “**” means extremely significant difference (*p* < 0.01).

At the concentration of 10 μg/μL, the attractive rates of the five volatiles to *B. terrestris* were not significant, and the attractive rates of e-2-hexenal and 2,5-dimethyl-benzaldehyde were higher than the repellent rates (*p* > 0.05). At the concentration of 200 to 500 μg/μL, the repellent rate of e-2-hexenal was significantly higher than the attractive rate (*p* < 0.05). At 100 μg/μL and 400 μg/μL, *B. terrestris* preferred liquid paraffin to e-2-octenal (*p* < 0.05), while other concentrations were not significant. At the concentration of 100 to 500 μg/μL, *B. terrestris* preferred liquid paraffin to benzaldehyde (*p* < 0.05). At 400 μg/μL, benzenepropanal was not as popular as liquid paraffin (*p* < 0.05). Compared with 2,5-dimethyl-benzaldehyde, *B. terrestris* preferred liquid paraffin (*p* > 0.05). At the same concentration of different volatiles, from 10 to 500 μg/μL, the volatiles with the highest attractive rate to honeybees were: e-2-hexenal (53.33%), 2,5-dimethyl-benzaldehyde (43.33%), e-2-octenal (36.67%), 2,5-dimethyl-benzaldehyde and e-2-octenal (33.33%), 2,5-dimethyl-benzaldehyde (36.67%), and 2,5-dimethyl-benzaldehyde (40%) (Figure 6).

#### 3.3.3. Comparison of Behavior Tests between *A. mellifera* and *B. terrestris*

**Figure 7:**
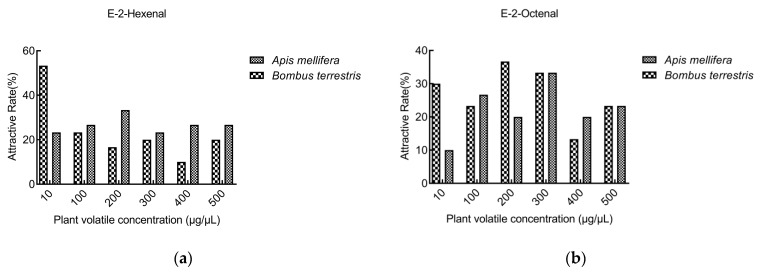
The attractive rate of compounds to *A. mellifera* and *B. terrestris*. (**a**) e-2-hexenal; (**b**) e-2-octenal.

Both e-2-hexenal and e-2-octenal were used in behavior tests of *A. mellifera* and *B. terrestris*. The attractive rate of e-2-hexenal to *B. terrestris* at 10 μg/μL (53.3%) was higher than that of *A. mellifera* (23.3%), but at other concentrations, *A. mellifera* was higher. The maximum attractive rate of e-2-hexenal to *B. terrestris* was at a low concentration of 10 μg/μL, but to *A. mellifera* was found at 200 μg/μL (Figure 7a). E-2-octenal was more attractive to *B. terrestris* than *A. mellifera* at concentrations of 10 μg/μL and 200 μg/μL. E-2-octenal had the maximum attractiveness to *B. terrestris* at 200 μg/μL and *A. mellifera* at 300 μg/μL (Figure 7b).

## 4. Discussion

In the nature, pollinators are attracted or shunted by factors such as the scent, shape, and color of flowers [16]. Floral fragrances are secondary metabolites released by flowering plants and are composed of volatile organic compounds (VOCs) mixed in varying proportions [17]. Volatiles released by flowers can influence the pollination behavior of pollinators such as bees [10,18,19,20]. The flower smells mainly include fragrant and malodorous types; however, both of these can help pollinate plants. For example, honeybees and butterflies can identify floral aromas to find reward food, and fetid odors will attract flies or beetles [8,18,20,21,22].

### 4.1. Identification of Volatile Compounds from Melon Flowers

In this study, the volatiles of male and female melon flowers were analyzed by SPME and GC-MS. A total of 77 compounds in 10 groups were detected in the female and male flowers of melon: ethers, alkynes, alkanes, alkenes, esters, aldehydes, alcohols, aromatics, ketones, and others. Among these volatiles, aldehydes, such as benzaldehyde and e-2-hexenal, had the most kinds and the highest relative content. Aldehydes are ubiquitous in flowering plants and play an important role in helping insects locate plants, avoiding pests, and attracting pollinators [23]. The relative content of benzaldehyde in female and male melon flowers was the highest (48.83%; 74.39%). Benzaldehyde is a common volatile organic compound and is the main component of flower fragrance in several crop plants [24], with an extremely complex chemical structure and a special aroma, mostly found in plant stems, leaves, and seeds. Fernandes identified both male and hermaphrodite flowers of five commercial types of melon *Cucumis melo* (Cantaloupe, Charentais, Galia, Piel de Sapo, and Yellow) and found only 1–2 aldehydes, of which benzaldehyde was identified only in Cantaloupe and Galia, and benzaldehyde was the most abundant among all volatiles in Cantaloupe hermaphrodite flowers [25]. The contents and types of volatile compounds in melon flowers of these varieties are different, which may be due to the genotypes, or environmental factors such as temperature and humidity, soil, water conditions, analysis conditions of volatile compounds, and precision of GC-MS instruments [26].

### 4.2. EAG Response of Bees to Volatiles

Examination of the EAG responses of *A. mellifera* and *B. terrestris* to the volatiles of male and female melon flowers showed that all the volatiles could induce electrophysiological responses of both species in different concentrations.

*A. mellifera* showed a strong EAG reaction to e-2-hexenal, e-2-octenal and 1-nonanal. *B. terrestris* showed a strong EAG reaction to benzaldehyde, e-2-hexenal, e-2-octenal, 2,5-dimethyl-benzaldehyde and benzenepropanal. In previous studies, 1-nonanal induced a strong EAG response to *A. mellifera* and *B. terrestris* [27,28,29]. In addition, 1-nonanal also causes EAG reactions in many insects, e.g., *D.helophoroides*, *A. glabripennis, B. horsfieldi*, and female adults of *Cydia molesta* [30,31,32,33]. E-2-octenal can induce a strong EAG response in *Tessaratoma papillosa* (Drury) adults [34]. Benzaldehyde can cause a strong EAG reaction in female adults of *Cydia* *molesta* [33]. E-2-hexenal can cause a strong EAG reaction in female *Bactrocera dorsalis* [35]. These results suggest that aldehydes, as volatile compounds of aromatics odors, may play a key role in insect olfactory recognition and selection.

The olfactory system of *A. mellifera* and *B. terrestris* is sensitive, and they can show different responses to different concentrations of the same volatiles. The level of odor concentration has a great influence on the electrophysiological responses of the two bee species. In this experiment, the electrophysiological responses of the two bee species to the volatiles of melon flowers showed different trends. The rEAG of *A. mellifera* to benzaldehyde, e-2-hexenal and e-2-octenal decreased with the increase in the concentration. The relative EAG response of *A. mellifera* and *B. terrestris* to 1-nonanal increased with the increase in the concentration. The recognition of odor by *A. mellifera* and *B. terrestris* would be affected by the concentration of volatile substances, possibly because the electrophysiological response of *A. mellifera* and *B. terrestris* has a certain threshold [36]. The optimal response concentration of *A. mellifera* and *B. terrestris* to different volatiles is different.

*A. mellifera* and *B. terrestris* on the same concentration of different volatiles have different EAG responses, possibly because of the different physical properties of volatiles, such as the different volatilization rate, which leads to the different time required for antennae to receive chemical signals. It is also possible that honeybees differ in their sensitivity to these volatiles [37], such as honeybees being more sensitive to 1-nonanal than other odors [27].

When the antennae of bees were stimulated by the same volatiles, the EAG response induced by some volatiles was stronger in *B. terrestris* than in *A. mellifera*, while others were just the opposite. This is possibly caused by the difference in the physiological structure of smell and smell sensitivity between *A. mellifera* and *B. terrestris* [37,38].

### 4.3. Behavior Tests

Behavior tests with the volatiles which could induce the strong EAG response of *A. mellifera* and *B. terrestris* showed the reaction of *A. mellifera* and *B. terrestris* to different concentrations of volatiles and showed different degrees of attraction and repellency.

The EAG response of *A. mellifera* to e-2-hexenal was strongest at 200 μg/μL, and the attractive rate (33.33%) was higher than the repellent rate (30.00%) (*p* > 0.05). The EAG responses of *A. mellifera* to e-2-octenal and 1-nonanal were the highest at 400 μg/μL, but the attractive rate to liquid paraffin was higher. In the behavior test of *B. terrestris*, at the concentration of 10 μg/μL, the attractive rate of e-2-hexenal and 2,5-dimethyl-benzaldehyde was higher than the repellent rate (*p* > 0.05). However, the EAG responses to e-2-hexenal and 2,5-dimethyl-benzaldehyde were the strongest at 100 μg/μL and 300 μg/μL, respectively. The electrophysiological response is not always presented in the behavioral response, and the phenomenon of inconsistency between the EAG results of antennae and the behavioral response often appears in the study of insect olfactory behavior [39,40,41]. It is possible that monomer compounds cannot cause directional reactions, and the combined effects of various odor volatiles should be considered [42,43]. The EAG response of all neurons in the antenna to the compound was measured by the potentiometer, and the response of the sensors in the antenna to the compound was measured by a monocesthesia recorder. Compared with the former, the EAG response values measured by the monocesthesia recorder were more similar to the results of the behavioral experiment [44]. The behavior selection of volatiles by insects is affected by many factors, such as environmental factors and their physiological conditions, rather than by a single active substance, which may be the main reason for the different results.

The attractive rate of e-2-hexenal to *B. terrestris* (53.3%) at 10 μg/μL was higher than that of *A. mellifera* (23.3%), but at other concentrations, the attractive rate to *A. mellifera* was higher. It suggests that the concentration of volatiles could affect the selection behavior of bees. The highest attractive rate of e-2-hexenal to *B. terrestris* was at the low concentration of 10 μg/μL, but to *A. mellifera* was found at 200 μg/μL. E-2-octenal had the maximum attraction to *B. terrestris* at 200 μg/μL and *A. mellifera* at 300 μg/μL. This may be caused by the difference in odor sensitivity between *A. mellifera* and *B. terrestris* [37]. Different volatiles or different volatile concentration can affect the behavior selection of bees, which is speculated to be one of the reasons why bees have a preference for different flowers. At the same time, different bee species have different preferences for flowers, which may also result in different bee species’ ability to collect the same crops. It was found that the appropriate concentration of 2,5-dimethyl-benzaldehyde and e-2-hexenal could attract *A. mellifera* and *B. terrestris*.

Insects are closely related to the volatiles of flowers, studies have shown that honeybees are attracted to flowers by flowers’ color and size, honeybees’ olfactory responses, and food rewards [45,46]. Insects may respond to visual stimuli or olfactory stimuli [12,47,48], while others may respond to both [49,50], e.g., honeybees. Moreover, odors were more attractive to honeybees than visual cues [12,51]. In our experiments, we collected and identified plant volatiles, of which we selected monomers and a variety of compounds to conduct electrophysiological and behavioral experiments, then screened out monomers with strong electrophysiological activity and mixed compounds in different proportions. However, since an EAG test can determine whether a single compound has reaction activity to insects and the optimal concentration range, which can reduce the range for the behavioral test and reduce blindness. Only a single compound was used to measure the EAG and behavioral responses of *A. mellifera* and *B. terrestris*. Moreover, the indoor behavioral environment is different from the natural environment. Therefore, compounds should be mixed in different proportions in the future to verify the pollination of *A. mellifera* and *B. terrestris* under greenhouse conditions, and to provide theoretical and technical support for the pollination of *A. mellifera* and *B. terrestris* in the facility cultivation of melon.

## 5. Conclusions

Floral fragrances play an important role in the pollination process of honeybees. Thirty-nine compounds (9 groups) and fifty-two compounds (10 groups) were isolated and identified from female and male melon flowers, respectively, among which aldehydes accounted for the most. Different concentrations of volatiles could induce EAG responses of *A. mellifera* and *B. terrestris*. *A. mellifera* showed strong EAG response to e-2-hexenal, e-2-octenal, and 1-nonanal. *B. terrestris* showed a strong EAG reaction to e-2-hexenal, e-2-octenal, benzaldehyde, 2,5-dimethyl-benzaldehyde, and benzenepropanal. In behavior tests, it was found that the appropriate concentration of e-2-hexenal and 2,5-dimethyl-benzaldehyde could attract *A. mellifera* and *B. terrestris*.

## Figures and Tables

**Table 1:** The standard compounds used in this study.

Compounds	CAS Number	Purity (%)	Origin
benzaldehyde	100-52-7	≥99.5	Aladdin
e-2-hexenal	6728-26-3	98	Aladdin
heptadecane	629-78-7	≥99	Macklin
benzenepropanal	104-53-0	95	Aladdin
trans-ß-ionone	79-77-6	>90.0	Aladdin
3-phenyl-2-propenal	104-55-2	98	Macklin
benzeneacetaldehyde	122-78-1	95	Aladdin
hexadecane	544-76-3	99	Macklin
1-nonanal	124-19-6	96	Aladdin
1,3-bis(1,1-dimethylethyl)-benzene	1014-60-4	>98.0	Aladdin
2,5-dimethyl-benzaldehyde	5779-94-2	98	Macklin
dimethyl phthalate	131-11-3	≥99.7	Aladdin
hexadecanoic acid, methyl ester	112-39-0	99	Aladdin
3-nonen-2-one	14309-57-0	≥96	Macklin
nonadecane	629-92-5	98	Macklin
2,2,4-trimethyl-1,3-pentanediol diisobutyrate	6846-50-0	98.5	Aladdin
e-2-decenal	3913-81-3	98.5	Aladdin
hexadecanoic acid, ethyl ester	628-97-7	≥99	Aladdin
e-2-octenal	2548-87-0	95	Aladdin
decanal	112-31-2	97	Aladdin
phytol	150-86-7	>90.0	Aladdin
1,2-benzene dicarboxylic acid, butyl octyl ester	84-78-6	95	Aladdin
dodecanal	112-54-9	95	Aladdin
1,2-dimethoxy-4-(1-propenyl)-benzene	93-16-3	>98.0	Aladdin

**Table 2:** Volatile compounds of female and male flowers.

Compounds	CAS Number	Female Flowers		Male Flowers	
		Retain Time (min)	Relative Content (%)	Retain Time (min)	Relative Content (%)
aldehydes					
benzaldehyde	100-52-7	7.952	48.83	7.938	74.39
e-2-hexenal	6728-26-3	4.566	7.13	4.619	3.22
benzeneacetaldehyde	122-78-1	11.268	1.06	11.203	1.06
1-nonanal	124-19-6	13.62	0.24	13.653	1.4
benzenepropanal	104-53-0	16.094	2.12	16.1	0.45
pentadecanal-	2765-11-9	32.562	0.4	34.003	0.04
2,5-dimethyl-benzaldehyde	5779-94-2	18.442	0.13	—	—
3-phenyl-2-propenal	104-55-2	21.706	1.38	—	—
retinal	116-31-4	33.622	0.05	—	—
decanal	112-31-2	—	—	17.961	0.19
2,4-dimethyl-benzaldehyde	15764-16-6	—	—	18.42	0.3
e-2-decenal,	3913-81-3	—	—	21.067	0.39
dodecanal	112-54-9	—	—	26.828	0.14
z-13-octadecenal	58594-45-9	—	—	30.962	0.09
hexadecanal	629-80-1	—	—	32.563	0.16
e-2-octenal	2548-87-0	—	—	11.737	0.26
alkanes					
2-methyltetracosane	1560-78-7	32.306	0.16	—	—
heptadecane	629-78-7	39.445	2.88	—	—
1,1,3-tricyclohexylpropane	55682-89-8	40.856	0.16	—	—
hexadecane	544-76-3	41.135	1.06	—	—
heneicosane	629-94-7	42.346	23.82	—	—
nonadecane	629-92-5	—	—	39.453	1.06
4-propoxy-4’-propyl-1,1’-bicyclohexyl	98321-58-5	—	—	40.627	0.03
tetracosane	646-31-1	—	—	42.333	4.6
ethers					
1,2-dimethoxy-4-(1-propenyl)-benzene	93-16-3	—	—	28.062	0.12
alkynes					
1-dodecyne	765-03-7	6.157	0.1	—	—
1-octadecyne	629-89-0	26.831	0.11	—	—
4-ethyl-3-nonen-5-yne	74685-67-9	—	—	23.908	0.85
alkenes					
11-chloro-1-undecene	872-17-3	26.079	0.27	—	—
3-ethyl-cyclohexene	2808-71-1	—	—	9.854	0.38
esters					
dimethyl phthalate	131-11-3	28.012	0.37	—	—
hexadecanoic acid, methyl ester	112-39-0	35.686	0.37	—	—
docosanoic acid, ethyl ester	5908-87-2	36.978	0.55	33.688	0.05
11-dodecen-1-ol trifluoroacetate	128792-46-1	40.712	0.3	—	—
1,2,4-benzene tricarboxylic acid, 1,2-dimethyl ester	54699-35-3	—	—	3.966	1.4
cyclohexanol, 2-methylene-3-(1-methylethenyl)-, acetate, cis-	54824-09-8	—	—	18.633	0.13
borinic acid, diethyl-, 1-ethynylcyclohexyl ester	55848-34-5	—	—	19.548	0.14
phenprobamate	673-31-4	—	—	22.921	0.34
pentanoic acid, 5-hydroxy-, 2,4-di-t-butyl phenyl esters	166273-38-7	—	—	29.335	0.39
2,2,4-trimethyl-1,3-pentanediol diisobutyrate	6846-50-0	—	—	30.719	0.53
1,2-benzene dicarboxylic acid, butyl octyl ester	84-78-6	—	—	34.843	0.14
hexadecanoic acid, ethyl ester	628-97-7	—	—	36.982	0.36
alcohols					
3,6,6-trimethyl-2-norpinanol	29548-09-2	25.545	0.28	25.552	0.43
2-methyl-6-methylene-2-octanol	18479-59-9	33.419	0.13	—	—
z,z-2,5-pentadecadien-1-ol	139185-79-8	33.724	0.06	—	—
3,7,11,15-tetramethyl-2-hexadecen-1-ol	102608-53-7	34.298	0.14	—	—
4-cyclooctene-1-methanol	13366-81-9	—	—	17.61	0.03
2-methyl-2-(4-methyl-3-pentenyl)-cyclopropanemethanol	98678-70-7	—	—	25.978	0.11
trans,trans-2,6-dimethyl-2,6-octadiene-1,8-diol	26488-97-1	—	—	26.083	0.09
2-butyl-1-octanol	3913-2-8	—	—	26.552	0.07
phytol	150-86-7	—	—	31.946	0.19
aromatics					
1,3-bis(1,1-dimethylethyl)-benzene	1014-60-4	20.486	0.15	20.486	0.3
2,4-di-tert-butylphenol	96-76-4	29.334	0.55	—	—
5-pentyl-1,3-benzenediol	500-66-3	29.459	0.62	29.466	0.15
asarone	2883-98-9	31.174	0.49	—	—
5-butyl-6-hexyloctahydro-1h-indene	55044-36-5	40.623	0.01	—	—
2,3,6,7-tetrahydro-3a,6-methano-3ah-indene	98640-29-0	—	—	21.792	0.35
5-ethyl-5-methyl-2-phenyl-2-oxazoline	91875-70-6	—	—	23.518	0.05
butylated hydroxytoluene	128-37-0	—	—	29.18	0.05
ß-asarone	5273-86-9	—	—	31.175	1.64
1,2,3-trimethoxy-5-(2-propenyl)-benzene	487-11-6	—	—	32.133	0.18
ketones					
4-(2,6,6-trimethyl-2-cyclohexen-1-yl)-3-buten-2-one	6901-97-9	27.293	0.14	—	—
trans-ß-ionone	79-77-6	28.635	1.53	—	—
4-cyclohexylidene-3,3-diethyl-2-pentanone	313253-65-5	32.093	0.2	—	—
1-phenyl-1-propanone	93-55-0	16.33	1.01	16.346	0.6
(z)- 6,10-dimethyl-5,9-undecadien-2-one	3879-26-3	27.897	0.26	27.897	0.17
(r)-5,6,7,7a-tetrahydro-4,4,7a-trimethyl-2(4h)-benzofuranone	17092-92-1	29.56	0.27	29.567	0.24
6,10,14-trimethyl-2-pentadecanone	502-69-2	34.413	1.34	34.413	0.38
(1-oxa-2-aza-spiro [2.5]oct-2-yl)-phenyl methanone	2289-83-0	—	—	12.351	0.17
3-nonen-2-one	14309-57-0	—	—	20.208	0.03
2,4,4-trimethyl-3-(3-methylbutyl)cyclohex-2-enone	88725-82-0	—	—	24.81	0.19
α-ionone	127-41-3	—	—	27.294	0.14
4-(2,2,6-trimethyl-7-oxabicyclo [4.1.0]hept-1-yl)-3-buten-2-one	23267-57-4	—	—	28.636	1.59
others					
dicyclopentadiene diepoxide	81-21-0	23.921	0.74	—	—
3-methyl-2-(3,7,11-trimethyldodecyl) furan	166773-55-3	35.617	0.61	35.627	0.14
ethinamate	126-52-3	—	—	10.652	0.1

**Table 3:** Both female and male melon flowers contain volatile compounds.

Compounds	CAS Number	Female Flowers		Male Flowers	
		Retain Time (min)	Relative Content (%)	Retain Time (min)	Relative Content (%)
alkenes					
docosanoic acid, ethyl ester	5908-87-2	36.978	0.55	33.688	0.05
aldehydes					
e-2-hexenal	6728-26-3	4.566	7.13	4.619	3.22
benzaldehyde	100-52-7	7.952	48.83	7.938	74.39
benzeneacetaldehyde	122-78-1	11.268	1.06	11.203	1.06
1-nonanal	124-19-6	13.62	0.24	13.653	1.4
benzenepropanal	104-53-0	16.094	2.12	16.1	0.45
pentadecanal-	2765-11-9	32.562	0.4	34.003	0.04
aromatics					
1,3-bis(1,1-dimethylethyl)-benzene	1014-60-4	20.486	0.15	20.486	0.3
5-pentyl-1,3-benzenediol	500-66-3	29.459	0.62	29.466	0.15
ketones					
1-phenyl-1-propanone	93-55-0	16.33	1.01	16.346	0.6
(z)-6,10-dimethyl-5,9-undecadien-2-one	3879-26-3	27.897	0.26	27.897	0.17
(r)-5,6,7,7a-tetrahydro-4,4,7a-trimethyl-2(4h)-benzofuranone	17092-92-1	29.56	0.27	29.567	0.24
6,10,14-trimethyl-2-pentadecanone	502-69-2	34.413	1.34	34.413	0.38
alcohols					
3,6,6-trimethyl-2-norpinanol	29548-09-2	25.545	0.28	25.552	0.43
others					
3-methyl-2-(3,7,11-trimethyldodecyl) furan	166773-55-3	35.617	0.61	35.627	0.14

## Data Availability

All data generated or analyzed during this study are included in this article.

## Appendix A

**Table A1:** The relative EAG response values of *Apis mellifera*.

Compounds	Relative EAG Response Values (rEAG)					
	10 μg/μL	100 μg/μL	200 μg/μL	300 μg/μL	400 μg/μL	500 μg/μL
heptadecane	0.84 ± 0.33 Aabcdef	0.31 ± 0.08 Bcd	0.26 ± 0.07 Bc	0.17 ± 0.05 Bd	0.26 ± 0.07 Bb	0.44 ± 0.24 ABb
3-phenyl-2-propenal	0.23 ± 0.07 Bef	0.24 ± 0.06 Bcd	0.31 ± 0.09 Bc	0.18 ± 0.06 Bcd	0.29 ± 0.10 Bb	0.63 ± 0.15 Ab
hexadecane	0.38 ± 0.15 Aef	0.16 ± 0.04 Ad	0.15 ± 0.07 Ac	0.31 ± 0.16 Abcd	0.34 ± 0.15 Ab	0.52 ± 0.27 Ab
2,5-dimethyl-benzaldehyde	0.24 ± 0.15 Aef	0.84 ± 0.24 Abcd	0.81 ± 0.31 Abc	0.86 ± 0.33 Abcd	0.79 ± 0.41 Ab	0.84 ± 0.50 Ab
dimethyl phthalate	0.60 ± 0.14 Abcdef	0.30 ± 0.13 ABcd	0.25 ± 0.11 Bc	0.40 ± 0.10 ABbcd	0.21 ± 0.06 Bb	0.23 ± 0.06 Bb
hexadecanoic acid, methyl ester	0.45 ± 0.17 Adef	0.15 ± 0.05 Ad	0.34 ± 0.20 Ac	0.48 ± 0.28 Abcd	0.59 ± 0.36 Ab	0.53 ± 0.30 Ab
benzaldehyde	1.43 ± 0.59 Aabc	1.84 ± 0.82 Abc	1.86 ± 0.87 Abc	2.06 ± 0.85 Abc	1.86 ± 0.89 Ab	1.82 ± 0.88 Ab
e-2-hexenal	1.02 ± 0.30 Aabcdef	2.32 ± 0.76 Ab	2.42 ± 0.86 Ab	2.15 ± 0.87 Ab	2.15 ± 0.80 Ab	1.84 ± 0.74 Ab
benzenepropanal	0.66 ± 0.28 Aabcdef	0.86 ± 0.38 Abcd	0.62 ± 0.20 Ac	0.81 ± 0.33 Abcd	0.72 ± 0.26 Ab	0.75 ± 0.29 Ab
benzeneacetaldehyde	1.51 ± 0.55 Aab	1.45 ± 0.64 Abcd	1.24 ± 0.41 Abc	0.93 ± 0.34 Abcd	0.97 ± 0.44 Ab	0.92 ± 0.42 Ab
1-nonanal	1.58 ± 0.53 Aa	4.68 ± 2.13 Aa	5.17 ± 2.34 Aa	5.71 ± 2.47 Aa	5.59 ± 2.45 Aa	5.96 ± 2.48 Aa
1,3-bis(1,1-dimethylethyl)-benzene	0.50 ± 0.19 Acdef	0.41 ± 0.14 Acd	0.37 ± 0.09 Ac	0.49 ± 0.09 Abcd	0.47 ± 0.16 Ab	0.52 ± 0.15 Ab
trans-ß-ionone	0.36 ± 0.10 Aef	0.49 ± 0.18 Acd	0.48 ± 0.14 Ac	0.78 ± 0.23 Abcd	0.87 ± 0.33 Ab	0.65 ± 0.34 Ab
3-nonen-2-one	1.15 ± 0.30 Aabcde	1.57 ± 0.61 Abcd	1.57 ± 0.66 Abc	1.40 ± 0.60 Abcd	1.44 ± 0.50 Ab	1.24 ± 0.49 Ab
nonadecane	0.51 ± 0.31 Acdef	0.44 ± 0.28 Acd	0.30 ± 0.17 Ac	0.98 ± 0.44 Abcd	0.71 ± 0.39 Ab	0.49 ± 0.32 Ab
2,2,4-trimethyl-1,3-pentanediol diisobutyrate	0.32 ± 0.11 Aef	0.24 ± 0.12 Acd	0.44 ± 0.07 Ac	0.22 ± 0.09 Acd	0.29 ± 0.10 Ab	0.48 ± 0.20 Ab
e-2-decenal	0.29 ± 0.10 Bef	1.11 ± 0.32 ABbcd	1.32 ± 0.48 ABbc	1.47 ± 0.46 ABbcd	1.54 ± 0.44 Ab	1.76 ± 0.53 Ab
hexadecanoic acid, ethyl ester	0.18 ± 0.07 Af	0.18 ± 0.07 Ad	0.18 ± 0.06 Ac	0.15 ± 0.04 Ad	0.31 ± 0.11 Ab	0.34 ± 0.10 Ab
e-2-octenal	0.94 ± 0.45 Aabcdef	1.36 ± 0.59 Abcd	1.69 ± 0.74 Abc	1.92 ± 0.65 Abcd	2.05 ± 0.90 Ab	1.95 ± 0.70 Ab
decanal	0.29 ± 0.05 Aef	0.39 ± 0.12 Acd	0.43 ± 0.14 Ac	0.75 ± 0.27 Abcd	0.60 ± 0.30 Ab	0.80 ± 0.37 Ab
phytol	0.46 ± 0.16 Adef	0.56 ± 0.22 Acd	1.04 ± 0.49 Abc	1.04 ± 0.61 Abcd	1.15 ± 0.64 Ab	1.16 ± 0.84 Ab
1,2-benzenedicarboxylic acid, butyl octyl ester	0.90 ± 0.55 Abcdfe	0.53 ± 0.21 Acd	0.89 ± 0.45 Abc	0.85 ± 0.34 Abcd	0.86 ± 0.49 Ab	1.03 ± 0.61 Ab
dodecanal	0.42 ± 0.22 Adef	0.48 ± 0.22 Acd	0.41 ± 0.18 Ac	0.39 ± 0.18 Abcd	0.46 ± 0.12 Ab	0.37 ± 0.13 Ab
1,2-dimethoxy-4-(1-propenyl)-benzene	1.35 ± 0.83 Aabcd	1.60 ± 0.88 Abc	1.31 ± 0.91 Abc	1.72 ± 0.99 Abcd	2.14 ± 1.03 Ab	1.91 ± 1.06 Ab

Data in the table are mean ± SE. Lowercase letters mean the significance between different volatiles at the same concentration (same column), and uppercase letters mean the significance between the same volatiles at different concentrations (same row) (*p* < 0.05).

**Table A2:** The relative EAG response values of *Bombus terrestris*.

Compounds	Relative EAG Response Values (rEAG)					
	10 μg/μL	100 μg/μL	200 μg/μL	300 μg/μL	400 μg/μL	500 μg/μL
heptadecane	0.30 ± 0.09 Ac	0.17 ± 0.04 Ae	0.35 ± 0.12 Ac	0.38 ± 0.17 Acd	0.42 ± 0.22 Ac	0.14 ± 0.02 Ac
3-phenyl-2-propenal	0.54 ± 0.03 Abc	0.48 ± 0.17 Ae	0.42 ± 0.26 Ac	0.43 ± 0.16 Acd	0.58 ± 0.16 Ac	0.54 ± 0.21 Abc
hexadecane	0.41 ± 0.21 Abc	0.21 ± 0.09 Ae	0.26 ± 0.09 Ac	0.32 ± 0.12 Acd	0.37 ± 0.22 Ac	0.54 ± 0.29 Abc
2,5-dimethyl-benzaldehyde	0.30 ± 0.18 Bc	1.73 ± 0.63 ABbcde	2.35 ± 0.94 ABbc	2.50 ± 0.50 Abcd	2.50 ± 0.86 Abc	2.18 ± 0.77 ABbc
dimethyl phthalate	0.89 ± 0.21 Abc	0.56 ± 0.25 Acde	0.64 ± 0.32 Ac	0.67 ± 0.29 Acd	0.44 ± 0.15 Ac	0.65 ± 0.29 Abc
hexadecanoic acid, methyl ester	0.46 ± 0.15 Abc	0.25 ± 0.06 Ae	0.33 ± 0.12 Ac	0.30 ± 0.10 Acd	0.13 ± 0.03 Ac	0.34 ± 0.11 Abc
benzaldehyde	3.00 ± 1.99 Aa	2.83 ± 2.07 Abcd	3.58 ± 2.69 Ab	4.04 ± 2.77 Ab	3.71 ± 2.90 Aab	1.56 ± 1.26 Abc
e-2-hexenal	0.78 ± 0.38 Abc	2.85 ± 1.13 Abc	2.41 ± 1.13 Abc	2.63 ± 1.24 Abcd	2.70 ± 1.28 Abc	2.78 ± 1.25 Ab
benzenepropanal	0.88 ± 0.51 Abc	3.16 ± 1.72 Ab	2.81 ± 1.66 Abc	3.06 ± 1.72 Abc	2.88 ± 1.69 Abc	2.32 ± 1.29 Abc
benzeneacetaldehyde	0.84 ± 0.18 Abc	0.54 ± 0.21 Ade	0.38 ± 0.19 Ac	0.32 ± 0.06 Acd	1.11 ± 0.62 Abc	0.57 ± 0.06 Abc
1-nonanal	0.77 ± 0.46 Abc	1.40 ± 1.09 Abcde	1.44 ± 1.23 Abc	1.80 ± 1.49 Abcd	1.98 ± 1.45 Abc	1.97 ± 1.44 Abc
1,3-bis(1,1-dimethylethyl)-benzene	0.37 ± 0.09 Abc	0.21 ± 0.09 Ae	0.50 ± 0.24 Ac	0.63 ± 0.08 Acd	0.50 ± 0.09 Ac	0.59 ± 0.10 Abc
trans-ß-ionone	0.57 ± 0.29 Abc	0.32 ± 0.14 Ae	0.52 ± 0.22 Ac	0.19 ± 0.11 Acd	0.35 ± 0.12 Ac	0.28 ± 0.09 Abc
3-nonen-2-one	0.48 ± 0.13 Abc	0.26 ± 0.08 Ae	0.31 ± 0.12 Ac	0.26 ± 0.11 Acd	0.45 ± 0.09 Ac	0.46 ± 0.19 Abc
nonadecane	0.44 ± 0.16 Abc	0.26 ± 0.04 Ae	0.22 ± 0.08 Ac	0.33 ± 0.20 Acd	0.26 ± 0.05 Ac	0.15 ± 0.08 Ac
2,2,4-trimethyl-1,3-pentanediol diisobutyrate	0.36 ± 0.15 Ac	0.48 ± 0.26 Ae	0.36 ± 0.12 Ac	0.46 ± 0.10 Acd	0.45 ± 0.04 Ac	0.22 ± 0.13 Ac
e-2-decenal	0.37 ± 0.05 Abc	0.95 ± 0.42 Abcde	1.11 ± 0.59 Abc	1.20 ± 0.56 Abcd	1.41 ± 0.47 Abc	1.36 ± 0.62 Abc
hexadecanoic acid, ethyl ester	0.42 ± 0.08 Abc	0.62 ± 0.12 Acde	0.35 ± 0.22 Ac	0.22 ± 0.11 Acd	0.36 ± 0.11 Ac	0.30 ± 0.16 Abc
e-2-octenal	1.75 ± 0.81 Aab	6.02 ± 2.17 Aa	6.92 ± 2.70 Aa	7.03 ± 2.96 Aa	5.91 ± 2.78 Aa	7.33 ± 3.19 Aa
decanal	0.69 ± 0.33 Abc	0.80 ± 0.45 Acde	0.83 ± 0.37 Ac	1.66 ± 0.83 Abcd	0.73 ± 0.39 Ac	1.44 ± 0.41 Abc
phytol	0.31 ± 0.15 Ac	0.24 ± 0.15 Ae	0.27 ± 0.11 Ac	0.17 ± 0.04 Ad	0.54 ± 0.28 Ac	0.28 ± 0.08 Abc
1,2-benzenedicarboxylic acid, butyl octyl ester	0.41 ± 0.19 bAc	0.30 ± 0.11 Ae	0.38 ± 0.13 Ac	0.30 ± 0.20 Acd	0.50 ± 0.23 Ac	0.71 ± 0.35 Abc
dodecanal	0.18 ± 0.14 Bc	0.88 ± 0.57 ABbcde	0.96 ± 0.40 ABbc	1.20 ± 0.49 ABbcd	1.46 ± 0.74 ABbc	1.78 ± 0.61 Abc
1,2-dimethoxy-4-(1-propenyl)-benzene	0.30 ± 0.09 Bc	0.22 ± 0.12 Be	0.34 ± 0.19 ABc	0.55 ± 0.09 ABcd	0.66 ± 0.20 Ac	0.43 ± 0.09 ABbc

Data in the table are mean ± SE. Lowercase letters mean the significance between different volatiles at the same concentration (same column), and uppercase letters mean the significance between the same volatiles at different concentrations (same row) (*p* < 0.05).
